# La cuerda dulce – a tolerability and acceptability study of a novel approach to specimen collection for diagnosis of paediatric pulmonary tuberculosis

**DOI:** 10.1186/1471-2334-6-67

**Published:** 2006-04-04

**Authors:** Felicia Chow, Nora Espiritu, Robert H Gilman, Rosmery Gutierrez, Sonia Lopez, A Roderick Escombe, Carlton AW Evans, David AJ Moore

**Affiliations:** 1 Asociación Benéfica Proyectos en Informática, Salud, Medicina, y Agricultura (AB PRISMA), Lima, Perú; 2 Hospital Nacional Dos de Mayo, Lima, Perú; 3 Universidad Peruana Cayetano Heredia, Lima, Perú; 4 Wellcome Trust Centre for Clinical Tropical Medicine, Imperial College London, UK

## Abstract

**Background:**

Recent data demonstrate the utility of the string test for the diagnosis of sputum-scarce HIV-associated TB in adults. We hypothesized that, if well-tolerated by children, this simple tool might offer a breakthrough in paediatric TB diagnosis. Thus the objective of this study, undertaken in the paediatric service of the Hospital Nacional Dos de Mayo, Lima, Perú, was to determine the tolerability and acceptability of the string test to paediatric TB suspects, their parents and nursing staff.

**Methods:**

22 paediatric subjects aged 3–14 years (median 8) under investigation for TB were invited to undergo 2 string tests (four-hour downtime each). Subjective and objective pain and discomfort rating scales were used to assess the perception of the subject, parent and attending nurse.

**Results:**

Patients as young as 4 years tolerated the procedure extremely well with 84% willing to undergo a second procedure. Peak discomfort at the time of swallowing and of string retrieval was mild (30% of maximum possible score) and brief as judged by visual analogue ratings and objective indicators. Good concordance of parent/child and objective/subjective ratings strengthened the validity of these findings.

**Conclusion:**

The string test is well tolerated and achievable for most paediatric TB suspects as young as 4 years. A formal prospective paediatric efficacy study is now needed.

## Background

Paediatric tuberculosis (TB) has long been neglected despite annual estimated morbidity of over 880,000 cases globally [1,2]. This partly reflects a global strategy (DOTS) focused upon transmission reduction in which children are deemed to play a small part and partly the notorious difficulty of diagnosing tuberculosis in this population, factors which are clearly related.

Paediatric TB suspects are frequently unable to spontaneously produce adequate sputum specimens, and samples tend to be paucibacillary. The poor sensitivity of even optimal current approaches yield a microbiological diagnosis in only 40% of children with tuberculosis disease [3,4].

The inadequacy of current diagnostics for pediatric tuberculosis dictates that the inherently unsatisfactory standard practice is empirical treatment based on clinical and epidemiological factors [5]. Ideally because of the difficulty of obtaining specimens from pediatric patients, these precious samples should be cultured but the benefits of greater sensitivity and specificity are conventionally diminished by delays of three to eight weeks. Using the rapid, inexpensive microscopic observation drug susceptibility assay (MODS) [6,7] reliable culture and susceptibility results are returned in a median of 9 days [8]. Inadequacy of respiratory secretion samples remains problematic. Gastric washings and nasopharyngeal aspiration may be used [9,10], but neither procedure is well tolerated. HIV-infected adults with tuberculosis co-infection, like children, often have difficulty producing sputum. We have recently demonstrated the superiority of the string test over sputum induction in this patient group; thus *M tuberculosis *was detected by culture using the string test in 15 of 160 HIV+ adult TB suspects, of whom only nine had positive induced sputum cultures (p = 0.03) [11]. The string test, originally developed for the retrieval of enteric pathogens [12,13] and *Helicobacter pylori*, consists of a coiled nylon string inside a gel capsule. The string unravels through a hole in the end of the weighted capsule as it descends into the stomach and the capsule then dissolves in the stomach, allowing the string to become coated with gastro-intestinal secretions containing whatever pathogens are present. When the string is retrieved four hours later, the capsule and weight have detached and are digested or passed unnoticed in the faeces.

A paediatric string test has previously been used in children for the detection of enteric pathogens [12,14-16], diagnosis of gastroesophageal reflux, confirmation of contaminated small-bowel syndrome and assessment of neonatal cholestasis [16-21]. To our knowledge, it has not been previously used in the diagnosis of tuberculosis in children nor has tolerability previously been investigated.

If well tolerated, the string test combined with MODS or other sensitive, rapid diagnostic tests such as MGIT or the Griess method could represent a significant step forward in our ability to diagnose tuberculosis in children. As the preliminary stage of a process to evaluate the efficacy of the string test in the diagnosis of pediatric tuberculosis, our objective was to investigate the tolerability and acceptability of the string test in children undergoing investigation for tuberculosis.

## Methods

### Sites and subjects

Subjects were recruited and procedures performed at Hospital Nacional Dos de Mayo (Lima, Perú) between July and September 2003; cultures were performed at Universidad Peruana Cayetano Heredia (Lima). Children between three and seventeen years attending outpatients, the emergency department or admitted to the paediatric ward, *and *undergoing investigation for possible pulmonary tuberculosis were invited to participate. Paediatric contacts of infectious adults under evaluation for tuberculosis were also invited to participate. Children receiving tuberculosis treatment or prophylaxis were ineligible. Usual medical care was unaffected by study participation.

### Procedure – clinical

The parent(s)/guardian(s) of all eligible children were invited to join the study, which was explained to both parent/guardian and subject; consenting parents/guardians read and signed an information sheet and consent form. Written assent of children over seven years, indicating their independent willingness to participate in the study, was also required.

Children then underwent the first of two string tests using string capsules made in-house for <$0.20 each, with market-purchased gelatin capsules. All procedures were performed early in the morning after an overnight fast. The procedure method evolved during the course of the study (see Results). Once swallowed, the trailing string was taped to the subject's cheek, remaining in-situ for four hours prior to controlled removal by the study nurse unless earlier removal was requested. Children were occupied and distracted with quiet activities (puzzles, drawing etc.) during the "down-time" to minimize irritation and the opportunity for inadvertent or deliberate string removal. None of the study nurses had any prior experience of administering the string test but followed the simple instructions and easily learnt how to encourage the children and achieve successful outcomes.

The course of the procedure was divided into 9 time periods (table 1). The views of the subject, parent(s)/guardian(s) and attending nurses were recorded in three separate straightforward semi-structured questionnaires at each timepoint.

**Table 1:** Objective and subjective scale scores by time period of string test procedure

Time period and description	Number of procedures with data	Subjective		Difference between parental and child scores ^$^	Objective	
				
		Mean child rating * (0 – 5)	Mean parental rating^# ^(0 – 5)		Mean behavioural pain scale ^§ ^(out of 10)	Mean objective pain scale ^§ ^(out of 10)
(1) before swallowing the capsule	39	0.51	1.51	p = 0.001	0.18	0
(2) at the time of swallowing of the capsule	35	1.58	2.13	NS	1.3	0.63
(3) 10 minutes after swallowing the capsule	32	0.29	0.73	NS	0.61	0.71
(4) one hour after swallowing the capsule	32	0.24	0.39	NS	0	0.19
(5) two hours after swallowing the capsule	32	0.39	0.63	NS	0	0.47
(6) three hours after swallowing the capsule	30	0.31	0.29	NS	0	0.13
(7) at the time of removal of the string	32	1.60	1.86	NS	2.61	1.03
(8) immediately after removal of the string	32	0.73	1.09	p = 0.01	0.25	0.28
(9) 30 minutes after removal of the string	31	0.13	0.35	NS	0	0.07

* Wong-Baker FACES Pain Rating Scale – 0 = very happy face, 5 = very sad face

# Linear visual analogue scale – 0 = no distress, 5 = very distressed

$ Student's paired t test

^§ ^As recorded by study nurse – 0 = no pain, 10 = maximum pain

*Subjects *indicated their distress/discomfort using the Wong-Baker FACES Pain Rating scale [22] (0 = Very Happy Face, 5= Very Sad Face). Open questions were also used and post-procedure subjects were encouraged to describe to an imaginary friend waiting for a string test how best to prepare for it.

*Parents/guardians *used a visual analogue pain scale correlated with the Wong-Baker Scale (0 = No distress, 5 = Very distressed), and also indicated their own distress at each timepoint. They were asked to report the ease with which the capsule was swallowed, whether the test was excessively or unnecessarily uncomfortable for the subject, and how tolerability might be improved.

*Nurses *used the Behavioral Pain Assessment Scale (Campbell, Detroit Medical Center 2000), assigning scores of 0–2 in the categories of Face, Restlessness, Muscle Tone, Vocalization, and Consolability (0/10 = no pain, 10/10 = maximum pain). Concurrently an Objective Pain Scale [23] was completed with a 0–2 score given in the categories of Percentage Change in Blood Pressure, Crying, Movement, Agitation and Verbal Evaluation or Body Language (0/10 = no pain, 10/10 = maximum pain).

If the subject and parent(s)/guardian(s) agreed to a second test, the procedure was repeated.

### Procedure – laboratory

Removed strings were transported to the laboratory within three hours in 15 ml Falcon tubes containing 1.5 ml 0.9% saline. Secretions adsorbed onto the string were eluted; this sample was decontaminated with NaOH-NALC. All samples were cultured in Löwenstein-Jensen and MODS, the latter as described previously (Caviedes, 2000).

### Ethical approval

Ethical approval was granted by Universidad Peruana Cayetano Heredia (UPCH), Asociación Benéfica PRISMA (AB PRISMA), Imperial College London, DISA Lima-Ciudad (Ministry of Health) and the Hospital Nacional Dos de Mayo.

## Results

### Study population and participants

The participation of 23 subjects was invited through their parent(s)/guardian(s), of whom one declined. One recruited subject refused to attempt to swallow the capsule. Thus it was possible to attempt to conduct at least one test in 21 of 22 (95%) eligible subjects. Median participant age was eight years (range 3 to 14); 55% were female. All subjects were undergoing investigation for possible pulmonary tuberculosis, 10 (48%) as in-patients and 11 (52%) as outpatients; 14 (67%) had a history of known tuberculosis contact, 12 of whom were symptom-free.

### Procedures

**Figure 1:**
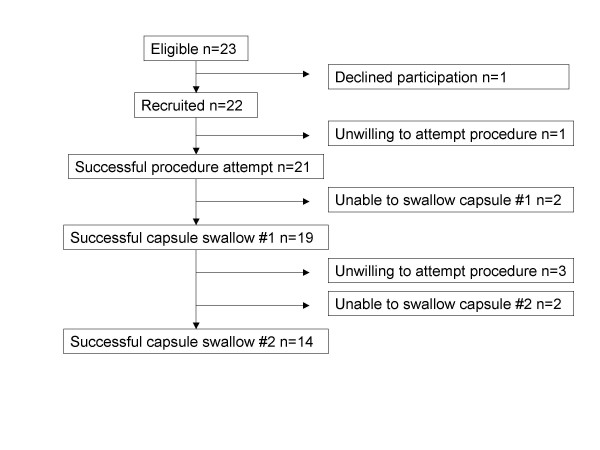
Flowchart of recruitment and study participation.

We aimed to perform the procedure twice (coherent with the two sputum samples required by the Peruvian National TB Programme). As an index of tolerability only 16% of subjects who successfully completed the first procedure declined the second. Two subjects were unable to swallow the capsule at all, two subjects were only able to swallow the capsule for the first test and three others were unwilling to attempt the second test. Thus we report here upon the results obtained in the course of 33 procedures undertaken by 19 subjects (figure 1).

In accordance with the adult protocol in use at Hospital Nacional Dos de Mayo, a four-hour "downtime" was required. Of the 33 tests performed, the down time for every test was the full four hours except in three instances. Two of these were self-removal by the patient at two hours (one inadvertent, one deliberate); the third was an inpatient admitted with vomiting, who successfully swallowed the capsule but vomited it back up within five minutes.

### Evolution of the procedure

Adults swallow the capsule with water, but to enhance the tolerability of the procedure for children we hypothesized that the use of a "sweetener" might be helpful ("la cuerda dulce" is Spanish for "the sweet string"). The study protocol allowed iterative evolution of the procedure method, in an effort to optimize participant tolerability. Embedding the capsule in a spoonful of flavoured jelly failed and was abandoned because of partial capsule dissolution, impairment of swallowing and because unraveling of the string from the descending capsule was impeded. Greater success was achieved by swallowing using either water alone or with a spoonful of honey followed by the capsule with water.

Once *in-situ *the drinking of warm liquid jelly helped to alleviate any discomfort from the string in the throat. Of the 33 successful swallows, 23 used water alone while 10 used honey and water.

### Rating of procedure overall

**Figure 2:**
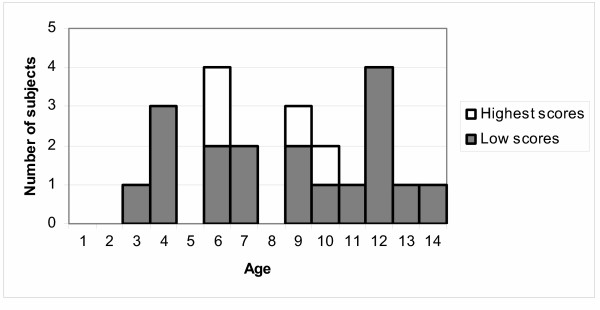
Participant age distribution and highest scoring quintile. Participants were grouped into quintiles according to overall score – individuals in the highest scoring quintile (n = 4) tolerated the procedure least well and are shown, by age, in white.

The procedure was very well tolerated as evidenced by overall median scores (median for the overall average score for each of the 9 time points) of 0 for both the subjective scales and both the objective pain scales. Tolerability scores were unrelated to age with four subjects (aged 6, 6, 9 and 10) disproportionately contributing to the overall mean scores (figure 2).

Few parents felt that improvements in tolerability were necessary; 92% reported not feeling upset or bothered at any point during the procedure.

Eighty-four percent of patients indicated a willingness to repeat the test the following day; 92% of parents said that they would be willing to persuade their child to repeat the test the next day. The youngest age that parents thought a child would be able to manage the string test varied from two and a half to six years.

**Figure 3:**
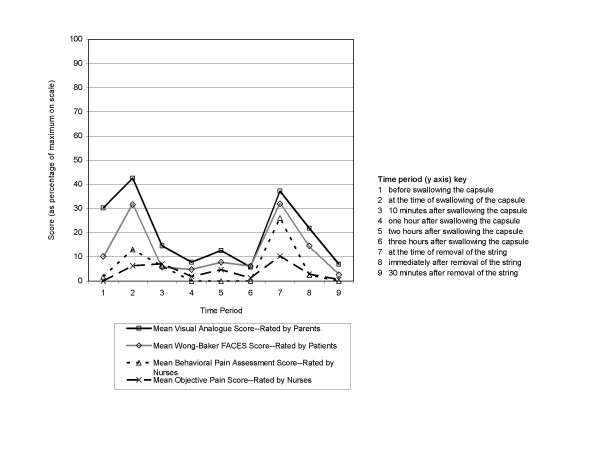
Mean objective and subjective scores for all patients at each time point.

Suggestions offered by subjects to a hypothetical friend included remaining calm and co-operative, keeping still during removal, not being nervous, and thinking positively. Although the peak mean FACES score was recorded at the time of swallowing, subjects described swallowing the capsule as "fine," "fun," "easy," "not at all terrible," "not painful," and "normal." Whilst the overall acceptability of the procedure was encouraging, the division of the procedure into nine distinct time-periods enabled us to identify the key moments of difficulty for closer scrutiny (figure 3).

### Time-course of the procedure (figure 3)

Of the nine time periods subjects, parents and nurses all agreed that most difficulty was encountered at time points 2 (capsule swallowing) and 7 (string removal).

#### Subjective assessment

The table shows the mean scores reported for subjects and their parent/guardian for each of these time points. Paired analyses revealed no significant differences between parental and subject responses except for the pre-procedure and immediate post-procedure periods when parents rated their children as more distressed than the children themselves.

Subjects' descriptions of string removal varied; most reported feeling fine, that nothing bad happened and that there was no worst part of the removal; some reported a tickling, pricking or scraping sensation in the throat; a few described the removal as if they were being choked and couldn't breathe, or that they felt nauseated. Many reported feeling afraid during the removal.

#### Objective assessment

The overall mean distress/discomfort scores for the whole procedure as assessed by the two objective scales were 0.6 and 0.4 (out of 10). Figure 3 illustrates two key points – firstly, outside of the two time points identified by subjects and parents alike as being the most difficult, the test caused virtually no distress; and secondly that the information provided by these objective measures corresponded well with that reported by the child participants and their parents (table 1).

The mean scores on the Behavioural Pain Assessment Scale and Objective Pain Scale (out of 10) were 1.3 and 0.6 at the time of swallowing the capsule and 2.6 and 1.0 at the time of string removal respectively, indicating only mild distress. Scores returned almost to baseline within 10 minutes of swallowing and immediately after string removal, indicating a very short duration of distress.

### Perceived ease of procedure

In general, parents and nurses rated the procedure as straightforward. Using a visual analogue scale (0 = Very Easy, 5 = Very Difficult) 77% and 63% of procedures were rated as 0, 1 or 2 by nurses and parents respectively. The capsule was successfully swallowed at the first attempt in 15 of the 33 successful procedures, on the 2^nd ^to 5^th ^attempt in 16 procedures and after more than five attempts in two cases.

Fear of string removal and associated discomfort and gag provocation associated were the reasons given for three subjects declining a second test. The youngest of the four patients unable to swallow the capsule was aged three and had never previously swallowed a capsule; though co-operative she chewed the capsule instead of swallowing it. The second youngest was a four year-old to whom the capsule was offered embedded within spoonfuls of solid jelly. She separated the capsule from jelly using her tongue, swallowed the jelly and retained the capsule in her mouth.

### TB diagnosis

There were no positive cultures for tuberculosis from any of the subjects, TB suspects or contacts, in this study.

## Discussion

The results of this study indicate that the string test is acceptable to and well tolerated by the majority of children down to the age of four years. The brief moments of capsule swallowing and string retrieval were least well tolerated but recovery was rapid. That the majority of participants were happy to undertake the test a second time reflects how relatively benign they regarded the experience. In a separate community-based study in Lima using the string test for *Helicobacter pylori *in children, subjects reported the procedure as preferable to an injection (Unpublished data, Kevin Nemethy 2003). One limitation of this data is the lack of children under 3 years of age, in whom rates of TB, particularly the most severe military and meningitic forms of disease, are highest. Given the potential difficulties of the crucial step of capsule swallowing in these patients it is clear that these data cannot be extrapolated to this group.

To our knowledge this is the first formal evaluation of the tolerability and acceptability of any procedure for retrieval of respiratory specimens from children and of the use of the string test (for any reason) in children. Our findings are consistent with previous anecdotal reports [16,17,20].

The concordance of all four subjective and objective measures of distress validates these indices and strengthens our assertion that this procedure is highly acceptable to children, and importantly also to their carers and attendant nursing staff. One study nurse was responsible for procedure supervision and another for completing the objective scale scores – it is not possible to exclude the play of bias in the assessments though we believe the concordance of scores from the different sources suggests this was not relevant. The willingness of all three parties to engage in the procedure is key to its success.

Our study design allowed for evolution and optimization of the procedural methodology in the light of our experience. Though child-dependent it soon became clear that either swallowing with water or in honey ("la cuerda dulce") was superior to the use of solid jelly (our initial working plan).

As a novel approach to the detection of *Mycobacterium tuberculosis *in swallowed respiratory secretions the string test shows great promise for adults with unproductive cough [11]. In this study, designed to assess tolerability and acceptability and not efficacy, no gold-standard comparator procedures were performed. Nevertheless it was very disappointing that we failed to confirm a microbiological diagnosis in any participant, several of whom were treated empirically for TB. This may reflect the use of an unnecessarily harsh (NaOH-NALC) decontamination procedure from which it is accepted that only 5–10% of mycobacteria survive. The impact of this effect is likely to be greater in children than adults owing to lower bacillary loads at the outset, which would explain why we did not encounter similar problems in HIV-infected adults [11]. Subsequent efficacy studies in children will need to explore less astringent decontamination, as recommended for gastric aspirates [24] particularly since the MODS methodology includes an antimicrobial cocktail in the media which reduces the necessity for harsh decontamination.

Of the variety of methods for obtaining clinical specimens for bacteriological confirmation of paediatric tuberculosis, gastric aspiration to obtain swallowed bronchial secretions is the most commonly used [25-27]. Nasopharyngeal aspirates have been used successfully [28], and have the advantage over gastric aspirates (and the string test) that they can be performed at any time of day. Data on the acceptability of any of the methodologies is lacking though clinical experience indicate that they are poorly tolerated. The yields of bronchoalveolar lavage (beyond the resources of most high TB-burden settings), sputum induction and gastric aspiration are broadly comparable with some data suggesting a modest advantage in favour of sputum induction and gastric aspirate culture [9,10,25]. Since the string test outperforms sputum induction in HIV-infected adults [11], a comparative efficacy and acceptability study of sputum induction, gastric aspiration and the string test for the diagnosis of paediatric pulmonary TB is now needed.

## Conclusion

This work has clearly established that the string test methodology is well tolerated by children as young as four years of age and is highly acceptable to the children, their parents and their attending nursing staff. Whether this procedure can enhance our ability to achieve a microbiological diagnosis in children with pulmonary tuberculosis can now be tested in formal efficacy studies.

## Competing interests

The author(s) declare that they have no competing interests.

## Authors' contributions

The study protocol was developed by DAJM, RHG, NE, CAWE, and ARE. Study design and execution was substantially revised and facilitated by FC, DAJM, NE, RG and SL. Field work was entirely carried out by FC, NE, RG and SL. DAJM, FC, RHG, CAWE and ARE were involved in review of data, analysis and manuscript preparation and revision.

## Pre-publication history

The pre-publication history for this paper can be accessed here:


